# Stereotactic prostate focal reirradiation therapy for local recurrence: preliminary results of Hartmann Oncology Radiotherapy Group

**DOI:** 10.1259/bjro.20180027

**Published:** 2019-06-07

**Authors:** Nathaniel Scher, Olivier Bauduceau, Marc Bollet, Hanah Lamallem, Tomer Charas, Pascal Garaud, Denis Foster, Maher Fawzi, Mona Labidi, Alain Toledano

**Affiliations:** 1 Hartmann Radiotherapy Institute, Hartmann Oncology Radiotherapy Group, Levallois-Perret, France; 2 Rafael Institute Center for Predictive Medicine, Levallois-Perret, France; 3 Department of Radiation Oncology, MSKCC, New York, USA

## Abstract

**Objective:**

Our objective was to report our experience and to evaluate the feasibility and toxicity of focal salvage stereotactic body radiation therapy (SBRT) in patients with post-radiation local recurrence of prostate cancer.

**Methods:**

We retrospectively reviewed medical records of patients treated with Cyberknife ^®^ between October 2014 and April 2017 at our institution for a focal reirradiation delivered to the prostate/prostatic bed for local recurrence after radical or adjuvant radiotherapy. All patients underwent prostate biopsies at recurrence at the time of fiducial markers placement, had choline PET/CT and pelvic MRI. The treatment consisted in 36 Gy in six fractions delivered every other day. Post reirradiation toxicities were assessed according to the CTCAE v4 (*Common Terminology Criteria for Adverse Events*).

**Results:**

42 patients were treated with followed with a median follow-up of 21 months (range 3 – 31). 34 patients had biopsy proven recurrence. The initial treatment was radical prostatectomy and radiation therapy for 9 patients and radiation therapy alone for 33 patients. 23 patients from the group of prostate reirradiation had placement of rectal spacers. No Grade 4 or 5 toxicity were observed. 27 acute urinary events were recorded: 18 patients experienced Grade 1, 9 patients experienced Grade 2 toxicity and 1 patient experienced Grade 3 urinary toxicity, namely cystitis and/or dysuria. No Grade 2 or more digestive toxicity was observed. Rectal doses were significantly lower with rectal spacers.

**Conclusion:**

Salvage focal Cyberknife ^®^ seems feasible and show promising results.

**Advances in knowledge:**

SBRT for local prostate cancer recurrence after initial radiotherapy is well tolerated with short follow-up.

External beam radiotherapy (EBRT) remains one of the mainstays among therapeutic approaches for the treatment of localized prostate cancer. The use of modern EBRT with higher radiation dose results in better biochemical outcomes. Despite this, 22 to 69% of males who receive EBRT will develop biochemical recurrence (BCR), which most often precedes clinical recurrence by years.^1–5^ In the case of demonstrated intra prostatic failure, guidelines support the use of an androgen deprivation therapy as the standard of care for the majority of patients.^6,7^ Potential side-effects of this treatment significantly impact quality of life. The optimal time to initiate ADT is a major challenge and will necessitate the use of long term ADT,possibly for life. 20 to 30% of all recurrent cases are local, that could benefit from local salvage therapy.^5,8^ The main goal of salvage therapy is to cure. There is no consensus regarding the most appropriate management of prostate cancer recurrence. Guidelines recommend local salvage therapy in patients with positive prostate biopsy who presented less than 10 ng ml^−1^ PSA, have no metastases and a 10-year life expectancy.^6^ In case of demonstrated intra prostatic failure after primary external beam radiotherapy, several salvage treatment options are available including salvage brachytherapy, salvage cryotherapy, high-intensity focused ultrasound (HIFU), or hormonal ablation. Salvage brachytherapy offers the ability to target radioactive sources to treat locally recurrent disease. Salvage brachytherapy show very low rates of grade ≥3 or above toxicity and half of the patients being biochemically controlled in long term.^9^ The use of salvage prostatectomy and salvage cryotherapy is somewhat limited due to treatment-related toxicity.

Studies of external beam reirradiation have been published for various tumor sites^10–12^ but they have scarcely been reported for intra prostatic recurrence. Indeed, several reasons could explain the rarity of this treatment: risk for normal tissue complication after high radiation dose, frequent metastatic evolution and elderly age. The technical evolution of radiotherapy offers new opportunities to treat small target with high radiation dose. SBRT has dosimetric and radiobiologic advantages as a salvage treatment paradigm. To limit normal tissue toxicity in the reirradiation situation, the target volume is confined to the recurrent macroscopic tumor in most clinical situations without targeting larger areas of potential microscopic spread.

CyberKnife^®^ (Accuray, Sunnyvale, CA) was established in our institution in 2014 and has been used to treat prostate cancer local relapses that are not eligible for other treatment modalities. The aim of this study was to retrospectively report our clinical experience and evaluate the safety, feasibility and toxicity of salvage stereotactic body radiation therapy (SBRT) using CyberKnife^®^ in patients with post-radiation local recurrence of prostate cancer.

## Methods

### Patient selection

We retrospectively reviewed medical records of patients treated with salvage CyberKnife^®^ for a post-radiation prostate cancer local recurrence between October 2014 and April 2017 at Hartmann Radiotherapy Institute. We collected clinical, biological parameters and dosimetric data. Patient reported outcomes and toxicities of the treatment were also collected according CTCAE v4 for all patients. Primary radiation therapy followed guidelines for low risk prostate cancer without ADT, for intermediate risk prostate cancer with short term ADT, for localized high-risk prostate cancer with long term ADT and for postoperative external-beam radiation therapy after radical prostatectomy. Inclusion criteria for reirradiation were: (1) The presence of a single recurrence from prostate cancer; (2) Exclusion of any metastases (choline PET/CT); (3) Diagnosis of prostate recurrence was based on biochemical failure confirmed by imaging studies; (4) Interval between first diagnosis of prostate cancer and diagnosis of recurrent disease of was greater than ≥2 years; (5) No severe (CTCAE v4 grade ≥3) chronic late toxicity; (6) Provision of written informed consent about this experimental treatment; (7) Presentation of case to multidisciplinary uro-oncology clinic. At time of recurrence, all patients underwent prostate biopsies and fiducial markers placement. All patients from the group prostate reirradiation underwent rectal spacer placement if they were treated after July 2015 (start date of the spacer implantation technique and a total of 42 patients were reviewed. Exclusion criteria were patients with lymph nodes metastasis and/or distant metastasis at time of reirradiation and those who underwent another local salvage local therapy. Table 1 shows patient characteristics. This study was approved by our Institutional Committee on Human Research.

**Table 1.** Patient characteristics (*n* = 42)

Number of patients	Prostate (*n* = 33)	Prostate bed (*n* = 9)	Total (*n* = 42)
**Age**			
Mean	67.2	59.63	65.5
Median [range]	66.5 [56;77]	62 [49;66]	64 [49;77]
**OMS**			
0	28	9	37 (88%)
1	4	0	4 (10%)
2	1	0	1 (2%)
>2	0	0	0
**Initial PSA** (ng/ml)			
Median [range]	12.9 [3;120]	7.6 [5;15]	10.1 [3;120]
**PSA nadir** (ng/ml)			
Median [range]	0.5 [0.01;1.8]	0.6 [0.01;0.6]	0.3 [0.01;1.8]
**Initial Gleason score**			
6	8	2	10 (25%)
7	17	6	23 (58%)
8	4	1	5 (13%)
9	2	0	2 (5%)
**Initial disease category:** D’Amico group			
Low	5	1	6 (16%)
Intermediate	10	5	15 (36%)
High	18	3	21 (48%)
**Initial treatment:**			
ADT	28	6	34 (84%)
RT 3D	26	9	35 (83%)
RT IMRT	2	0	5 (12%)
brachytherapy	2	0	2 (5%)
**Initial RT dose:**			
Median [range] (months)	74 [70;76]	68 [65;70]	74 [65;76]
**Interval between initial RT and reirradiation**			
Median [range] (months)	65 [29;204]	128 [54;207]	82.5 [29;207]

### Planning and treatment (Tables 1 and 2)

Stereotactic reirradiation was delivered using the CyberKnife^®^ accelerator (Accuray, Sunnyvale, CA). For all patients, four gold fiducial markers were placed in the prostate or in the prostate bed via trans-rectal ultra-sonography for tracking modalities. Then, a non-contrast pelvic CT scan with 1.25-millimeter slice thickness were performed. The Gross Tumor Volume (GTV) was defined as the recurrence observed on multiparametric MRI and the delineation was guided by PET/CT images. GTV based on MRI T2 was delineated by a radiation oncologist. If the T2 image showed hypo intensity and PET/CT image also indicated a lesion at the same location, the region was considered positive. The presence of tumor recurrence on PET/CT images was defined as any monofocal uptake greater than adjacent background uptake in more than one slice within the CT defined prostate gland. To exclude background signalling, the bladder was delineated and excluded from GTV PET if necessary. Clinical Target Volume (CTV) was defined as GTV for prostate recurrence and GTV plus 1 mm margin for prostate bed recurrence. The Planning Target Volume (PTV) was defined as the CTV plus a 3 mm margin for prostate recurrence and CTV plus 1 mm margin for prostate bed recurrence. The following organs at risk were delineated on each slice: rectum, bladder, femoral heads, penile bulb, bowel, testicles. The stereotactic radiation therapy dose prescribed was 36 Gy on the 80% isodose line, in six fractions, three fractions a week. Normal tissue dose constraints for planning are for the rectum: V27 <20 cm3 and D max = 40.5 Gy ; and for the bladder V19 <15cm3 V40<5 cm3. Cyberknife^®^ imaging system was taking X-ray images each 45 sec in order to provide real-time information about the location of the prostate/prostate bed and enable the system to dynamically track and correct for any movement of the target. All four fiducials selected at the planning stage were actually tracked during each treatment session fraction. Table 2 2 shows treatment characteristics.

**Table 2.** Treatment characteristics (*n* = 42)

Number of patients	Prostate (*n* = 33)	Prostate bed (*n* = 9)	Total (*n* = 42)
**Pre reirradiation PSA**			
Median [range] (months)	3.3 [0.05;23.7]	1.1 [0.01;3.1]	3.1 [0.01;23.7]
ADT group	0,4 [0.05–1.1]	0,3 [0.01–0.07]	0,3 [0.01–1.1]
Non-ADT group	4 [2.5–23.7]	2.6 [2.2–3.1]	3,9 [2.2–23.7]
ADT added to reirradiation	5	3	8 (19%)
Median duration of ADT (months)	6	6.1	6
Median time from start of ADT to re irradiation (months)	3 [1-10]	3 [1-5]	3 [1-10]
Recurrence biopsy proven	28	6	34 (80%)
Pelvic MRI before reirradiation	33 (78%)	9 (22%)	42 (100%)
Rectal spacers	23	0	23 (55%)
**CyberKnife** ^®^ **data**			
Median total dose (Gy)	36	36	36
Dose/fraction	6	6	6
Number of fractions	6	6	6
Estimated duration of a fraction (mn)	30.7	28.8	30.3

### Dosimetric data

We retrospectively collected the dosimetric data for all patient for PTV, CTV and organs at risk (bladder and rectum) using Multiplan. Table 3 shows the dosimetry of 42 patients. We also evaluated the effect of the hydrogel rectal spacer on rectal dosimetry and the thickness of the rectal spacer at midgland.

**Table 3.** Dosimetric data (*n* = 42)

Number of patients	Prostate with rectal spacer (*n* = 23)	Prostate without rectal spacer (*n* = 10)	Prostate bed (*n* = 9)	Total (*n* = 42)
**PTV** (median)				
D min	31.3 [20 ; 38]	31.3 [22 ; 38]	33 [24 ; 35]	31.4 [20 ; 38]
D mean	29.8 [27 ; 41]	29.8 [27 ; 41]	40.2 [35 ; 42]	40.1 [27 ; 42]
D max	42 [31 ; 45]	42 [31 ; 45]	45 [39 ; 47]	45 [31 ; 47]
Volume (cm3)	33 [6 ; 61]	31 [5 ; 56]	6 [4 ; 14]	29 [4 ; 61]
**CTV** (median)				
D min	34 [4 ; 56]	31.3 [3 ; 51]	31.3 [20 ; 38]	34 [3 ; 56]
D max	41 [28 ; 43]	38 [28 ; 40]	40.1 [27 ; 42]	41 [28 ; 43]
D mean	46 [33 ; 48]	42 [31 ; 45]	45 [31 ; 48]	45 [31 ; 48]
PTV **CI^a^** (median)	1.1 [1.21 ; 1.38]	1.2 [1.1 ; 1.48]	1.35 [1.14 ; 1.4]	1.17 [1.1 ; 1.4]
PTV **nCI^a^** (median)	1.1 [1.1 ; 1.4]	1.2 [1.1 ; 1.5]	1.4 [1.19 ; 1.5]	1.21 [1.1 ; 1.5]
PTV **HI^a^** (median)	1.2 [1.1 ; 1.3]	1.2 [1.1 ; 1.4]	1.27 [1.23 ; 1.3]	1.25 [1.1 ; 1.3]
**Rectum**				
D 20%	8 [4.45 ; 24]	12 [6 ; 26.7]	9.9 [2.1 ; 25.7]	10 [2.1 ; 26.7]
D 50%	5 [3.02 ; 36]	8 [6 ; 41.9]	8.6 [0.4 ; 59.3]	7 [0.4 ; 59.3]
V 27 Gy (cc)	0.1 [0 ; 4.5]	1.1 [0.8 ; 8.5]	0.9 [0 ; 19.6]	0.7 [0 ; 19.6]
D max	27.9^9,13^	34.4 [11 ; 39.9]	34.4 [0 ; 41.4]	30.2 [0 ; 41.4]
**Bladder**				
D 5 cm3	22.5 [11.7 ; 42.8]	23 [11.5 ; 42.8]	15.9 [11.5 ; 20.3]	21.2 [11.5 ; 42.8]
V 19 Gy (cc)	8 [1.1 ; 19]	8,6 [1.5 ; 20]	4.4 [1.8 ; 9.1]	7.1 [1.1 ; 19.6]
D max (Gy)	38 [2 ; 41]	39 [26.2 ; 41]	36.6 [26.2 ; 44.4]	38.2 [25.2 ; 44.4]

a CI: conformality index, nCI; new conformality index, HI; homogeneity index

Follow up after salvage reirradiation:

After salvage reirradiation, each patient was seen at 6 weeks, 3 months and then every 6 months by a radiation oncologist to assess clinical toxicities and PSA level. The severities of acute (within 30 days of ending radiation) and late toxicities were evaluated according to the Common Terminology Criteria for Adverse Events (CTCAE) version 4.0. In this study, biochemical response to treatment was classified as a reduction, stabilization, or progression in the level of PSA. A substantial reduction in PSA level (>50% PSA of initial value) was considered as a complete biochemical response; a reduction between 10 and 50% of the initial value was considered as a partial response and if it oscillated within the 10% margins of the initial value, it was classified as a stabilization; an increase of more than 10% was considered as a progression. The initial PSA value was the last value measured before the reirradiation. The group of patients treated with neoadjuvant, concomitant or adjuvant ADT was excluded for the evaluation of biochemical response. All patients with biochemical failure underwent a choline PET/CT. Biochemical failure was defined according the Phoenix ASTRO criteria as a rise of PSA of at least 2 ng ml^−1^ above the nadir after EBRT.^14^ Clinical progression was classified as the development of the disease in field or out field or distant metastasis.

### Statistical method

The statistical comparisons were performed using Mann-Whitney test with estimation of the *p* value by Monte-Carlo method (1,0000 iterations). *p* values were two-sided and considered to be statistically significant if less than 0.05. Calculations were made with the use of Addinsoft ^©^ XLSTAT (2016 - Premium).

## Results patients

Between October 2014 and April 2017, 42 patients with an isolated recurrence of prostate cancer were included with a median follow up of 21 months (range 3–31) (Table 4). For the primary treatment, radical prostatectomy and radiation therapy was performed in nine patients and radiation therapy alone in 33 patients. The initial disease category according to D’Amico group was low for six patients, intermediate for 15 patients and high for 21 patients. The interval between the diagnosis of prostate cancer and the first day of CyberKnife^®^ treatment ranged from 29 to 204 months (median, 82 months). All patient had prostate biopsies at recurrence. 34 patients have pathological features of malignancy: 28 from the prostate group and eight from the prostate bed group. At time of diagnosis of local recurrence, all patients underwent a choline PET/CT and a multi parametric prostate MRI. In addition, 21 patients (50%) underwent a multi parametric prostate MRI the week before simulation CT and for them, MRI and CT images were registered with image fusion. eight patients have received a neoadjuvant and concomitant ADT associated with salvage therapy for a mean duration of 6 months. The median time from start of ADT to reirradiation was 3 months (range, 1 to 10). The median PSA level at the time of salvage therapy for patient who have received ADT were 0.4 [0.05–1.1] for prostate group and 0.3 [0.01–0.07] for prostate bed group. The median PSA level at time of salvage therapy for patient who have not received ADT were 4 [2.5–23.7] for prostate group and 2.6 [2.2–3.1] for prostate bed group. 23 patients from the group of prostate reirradiation had placement of rectal spacers. Median gel thickness at midgland was 6.8 mm, range [3.5–16.9].

**Table 4.** Treatment outcome (*n* = 42)

Number of patients	Prostate with rectal spacer (*n* = 23)	Prostate without rectal spacer (*n* = 10)	Prostate bed (*n* = 9)	Total (*n* = 42)
**Follow up duration**				
Median[range]	21 [3;31]	18 [4;28]	17 [3;20]	21 [3;31]
**PFS**				
Median [range] (months)	10 [3 ; 21]	9 [4 ; 20]	11 [3 ; 17]	11 [3 ; 21]
**Site of progression**				
**In field**	0	0	0	0
**Out field**	4	2	1	7 (17%)
-Bones	1	0	0	1 (2%)
-Lymph nodes	3	1	1	5 (12%)
-Bones and lymph nodes	1	0	0	1 (2%)
**Biochemical only**	0	0	1	1 (2%)

### Treatment

42 patients were treated: 33 from the prostate group and nine from the prostate bed group. No patients were excluded for dose constraints considerations. The median PTV volume was 33 cm3 (range, 5 to 61) and 6 cm3 (range, 4 ; 14) for prostate group and prostate bed group respectively. The dosimetric plan parameters are detailed in Table 3.

### Reponses

Biochemical response was evaluated for the 34 patients of the group who have not received ADT (Table 5). At last follow-up, a biochemical complete response was observed in 26 patients (76%); a biochemical partial response was noticed in three patients (9%). Biochemical stabilization was observed in three patients (9%). Two patients (6%) experienced a biochemical failure. All patients were alive at the last follow up. Biochemical failure occurs with a median time of 10 months after radiation therapy. None had a clinical local failure. Six patients had regionally recurrence with pelvic lymph nodes progression confirmed by choline PET/CT, two had a distant failure with bones metastasis and one had both (Table 4). No local recurrence in the prostate or in the prostate bed were observed on follow up images. At 21 months, biochemical complete response was observed in 25 patients (75%): six patients (100%) of prostate bed reirradiation and 19 patients (69%) of the other group.

**Table 5.** Toxicities according CTCAE v4 (*n* = 42)

Number of patients	Prostate with rectal spacer (*n* = 23)	Prostate without rectal spacer (*n* = 10)	Prostate bed (*n* = 9)	Total (*n* = 42)
**Acute urinary toxicity** **All:** Grade 1, Grade 2, Grade 3, > Grade 3 **Cystitis** Grade 1, Grade 2, Grade 3 **Dysuria** Grade 1, Grade 2 Others	14: 10, 3, 1, 0 13, 3, 1 4, 4 0	10: 6, 3, 0, 0 3, 1, 0 3, 3 0	4: 2, 3, 0, 0 2, 1, 0 0, 1 0	28 (64%) 21 (50%) 15 (36%) 0
**Acute rectal toxicity** **Diarrhea** Grade 1 > Grade 1 Others	0, 0 0	2, 0 0	1, 0 0	3 (7%) 0
**Late urinary toxicity** **Cystitis** Grade 1 Grade 2 > Grade 3 **Dysuria** Grade 1 > Grade 1 **Urinary incontinence** Grade 3 Others	2, 1, 0 3, 0 1 0	2, 0, 0 1, 0 0 0	1, 1, 0 0 0 0 0	7 (17%) 4 (9%) 1 (2%) 0
**Acute rectal toxicity** **Diarrhea** Grade 1 > Grade 1 Others	0, 0 0	2, 0 0	1, 0 0	3 (7%) 0
**Late rectal toxicity** **All**	0	0	0	0

**Table 6.** Biochemical response (*n* = 34)

Number of patients	Prostate (*n* = 28)	Prostate bed (*n* = 6)	Total (*n* = 34)
**Biochemical response at last follow up**			
Complete response	21 (75%)	5 (83%)	26 (76%)
Partial response	3 (11%)	0	3 (9%)
Stabilization	3 (11%)	0	3 (9%)
Progressive disease	1 (4%)	1 (4%)	2 (6%)
**Biochemical response median follow up (21 months**)			
Complete response	19 (69%)	6 (100%)	25 (74%)
Partial response	2 (6%)	0	2 (6%)
Stabilization	2 (6%)	0	2 (6%)
Progressive disease	5 (18%)	0	5 (15%)

### Toxicity

15 patients (36%) did not have any acute or late toxicity. No Grade four or five toxicity were observed. The toxicity is reported in Table 5. We recorded 28 acute urinary events: 18 patients (43%), nine patients (21%) and one patients (2%) experienced Grade 1, Grade 2 and Grade 3 urinary toxicity respectively, namely cystitis and/or dysuria. No bladder spasm, hematuria or urinary incontinence were reported. Acute dysuria was significantly correlated with D max of the bladder (*p* = 0.037, CI99% 0.032; 0.042). We observed 10 (24%) late urinary events: eight Grade 1, one Grade two and one Grade 3 (urinary incontinence). According CTCAE v4, we recorded three (7%) acute Grade one rectal toxicity corresponding to diarrhea without any rectorrhagia or other GI toxicities. No late gastro intestinal events were reported during the follow up period. We evaluate also the clinical tolerance and the effect of hydrogel rectal spacer on rectal dosimetry. There were no device-related adverse events, rectal perforations, serious bleeding, or infections following the placement of rectal spacers. Moreover, rectal doses were statistically lower with rectal spacer. We found a significant increase in the volume of rectum receiving high radiation dose without a rectal spacer as compared with a rectal spacer when treating with SBRT. The rectal V27 and D max were significantly lower with the rectal spacer. The median V27 was 0.1 cc with rectal spacer and 1.1 cc without, *p* value = 0.022, CI 99% [0.018; 0.026] and median D max respectively 27.9 Gy and 34.4 Gy, *p* value = 0.027, CI 99% [0.023; 0.031]. Functional success (7.5 mm space after hydrogel placement) and clinical success (≥25% reduction in rectal V27) was achieved in all of the patients. Acute or late rectal toxicities were equivalent between patient with and without rectal spacer.

## Discussion

There is no consensus on the most appropriate management of patient with recurrent prostate cancer after primary radiation. Several late toxicities about non-radiation-based salvage therapy have been reported, such as rectal fistula after HIFU.^4^ Moreover, there is no robust evidence in terms of disease control in favor of cryotherapy or HIFU.^4^ The evolution of imaging and treatment modalities has allowed extremely precise tumor targeting using extreme hypo fractionation. High fractionation sensitivity is an intrinsic property of primary prostate cancer. Recent *in vivo* and clinical data suggest that prostate cancer may benefit from hypo fractionation because its α/β ration is lower than that rectum and other pelvic organs.^5^

Preliminary evidence of reirradiation in other areas has started to emerge. Therefore, reirradiation has become part of clinical practice guidelines.^6^ SBRT is particularly interesting option because it makes possible to reduce the safety margin around the target and thus spare the exposure of the previously irradiated normal tissue. In this context, stereotactic focal reirradiation seems to be particularly attractive. The literature consists of small-sized series, making it difficult to assess and compare dose and fractionation. Reirradiation doses were variable ranging from 15 to 60 in three fractions. The median dose was 30 Gy in a median of 4.5 fractions.^11^ Our treatment regimen of 36 Gy in six fractions is equivalent to the regimen use is other studies in the same clinical setting.^15,16^ This regimen seems to be safe and to obtain good results in terms of efficacy.

In our series, two groups of patients were analyzed: reirradiation on prostate and reirradiation prostatic bed. Our intention was to report a clinical experience and to evaluate the feasibility of SBRT in patient with a single recurrence of prostate cancer in irradiated field. To date, only few studies on stereotactic focal reirradiation for a local recurrence of prostate cancer have been published. Zerini and al retrospectively evaluated 32 patients reirradiated to the prostate or prostatic bed for local recurrence with a dose of 25 Gy in five fractions.^17^ They have reported lower acute urinary toxicity than in our study (19% *vs* 43% of Grade 1 and 6 *vs* 21% of Grade 2) with no Grade three or more urinary toxicity. Moreover, they mention a 2 years tumor control in about half of the patients. Jereczek-Fossa reported 15 patients reirradiated for local recurrence and four patients reirradiated for anastomosis recurrence at a dose of 30 Gy in five fractions.^12^ They find respectively 33% of acute urinary toxicity in prostate group and 25% in prostate bed group with a single Grade three urinary acute and late urinary toxicity. 75% of patients had a complete biochemical response at the last follow-up which is close to the results found in this study. Moreover, in the retrospective study of Janoray and al, 21 patients were treated with 36.25 Gy in five fractions for local in field recurrence after radical or post prostatectomy radiotherapy.^15^ The 1-year biochemical recurrence free survival and local control rates were 83.3 and 90% respectively. At 21 months, we notice similar results with a biochemical free survival of 72%. Mbeutcha and al reported 18 patients reirradiated using focal SBRT with 35 Gy in five fractions.^16^ After a median follow up of 14.5 months, 10 out of the 18 patients (56%) remained free of biochemical recurrence and a single patient experienced transit Grade four complication. Our study showed that reirradiation seems to be effective and safe. Our results on urinary and digestive toxicities are comparable to those obtained by other salvage therapy. However, further prospective studies and long-term follow up are needed to confirm the rate of acute and late toxicity events. On the basis of data on the correlation between late and acute toxicities,^3,8^ we can expect a limited rate of late toxicities. However, a longer follow up is needed to confirm this hypothesis.

The low rectal toxicities could be related to the setup of rectal spacer for more than half of the patients. We found a significant increase in the volume of rectum receiving high dose radiation without a rectal spacer as compared with a rectal spacer. This increase in rectal dose and the implication for a potentially higher risk of rectal toxicity should be considered when deciding to use a rectal spacer. The hydrogel rectal spacer appears to be an effective tool, potentially enabling advanced prostate RT protocols. Prospective clinical studies are needed to identify clinical risk factors to select patients who are expected to benefit most from rectal spacer implantation.

The use of fiducials in modern radiotherapy is recommended especially when considering dose escalation.^18,19^ The use of fiducial markers for prostate/prostate bed daily localization and set up reduces required PTV margins. If fiducial tracking is not possible, we do not recommend reirradiation using spine tracking as it requires larger PTV margins exposing the patient to a risk of significant toxicity and recurrence. The use of rectal spacers and fiducials allow a reduction in acute rectal toxicity^20^ but there is a lack of data on late toxicity and larger long-term studies are required. Another approach of external beam salvage irradiation is to do a reirradiation of the whole prostate gland. Two studies^12,21^ report similar results as focal reirradiation in term of biochemical disease-free survival and toxicity. Nevertheless, Zili and al. show after a median follow-up of 94 months, that whole gland reirradiation result in a high rate of severe radiation induced side-effect and poor long term biochemical and local control.^22^ Our approach for reirradiation was slightly different as we used focal reirradiation instead of whole gland treatment. Given the evidence from pathology studies that recurrences are frequently localized at the site of the primary tumor,^23,24^ a focal salvage approach might be a viable treatment option for patients with unifocal prostate cancer recurrence without metastatic disease. Whole gland salvage techniques carry a high risk of toxicity^22^ thus a focal salvage approach might reduce the risk of adverse events while maintaining cancer control. In this study, no local relapse was observed after salvage SBRT. Focal SBRT could be an option to delay the introduction of ADT in some highly selected patients, without exposing patients to high risk of complication.

Low and high-dose rate brachytherapy has also been described as a salvage treatment following radiotherapy. The results of low dose rate salvage brachytherapy have been reported over the last 10 years. Studies have indicated that while tumor control can be achieved in 30 to 50% of patients, toxicity outcomes were increased possibly related to the use of less sophisticated planning techniques.^25^ Available data on salvage reirradiation with high dose rate brachytherapy are sparse and radiation protocol used are heterogeneous. To date, only one Phase II prospective study is available. Yamada et al treated 42 patients with a total dose of 32 Gy in four fractions. Survival without biochemical relapse at 5 years following high-dose-rate brachytherapy was 69% with a median survival time of 36 months; 15% of the patients presented Grade two toxicity, and one patient presented Grade three incontinence.^26^ Moreover, dosimetric comparison between stereotactic body radiotherapy and high dose rate brachytherapy found that EBRT was not able to achieve either the high doses to the prostate or the dose-sparing effect on normal tissues that HDR brachytherapy is able to achieve,^27^ showing the advantages of HDR over external beam techniques. However, because Cyberknife^®^ seems to be the most advanced and suitable technique for prostate EBRT, and an effective and well tolerated tool for the prostate reirradiation, future studies should compare clinical outcomes and toxicity between these modalities.

They are several limitations in this study. One of the main limitation is the heterogeneity of patients. We choose to include both prostate and prostate bed reirradiation because our purpose was to report our experience and the feasibility of this treatment. Otherwise, all patient had prostate biopsies at time of recurrence. Nevertheless, 20% of them have non-pathological features of malignancy with evidence supporting a local recurrence: PSA kinetic, multiparametric pelvis MRI and choline PET/CT findings. Choline PET/CT and MRI have respectively yielded excellent results to confirm focal disease.^28,29^ We choose to hold these patients eligible and we consider that these data are sufficient evidence for salvage reirradiation. Despite very good sensibility of multiparametric MRI and choline PET/CT, prostate biopsy remains the gold standard for diagnosing prostate cancer.^13^ The effective of our study is small and the follow-up is relatively short. Thus, it is complicated to assess the biochemical response and the late toxicity. Howerver, to our knowledge, it is still the longest and largest study published for now and we believe that early results are important in this pathology. The purpose is to report the preliminary result even if prospective studies are warranted.

## Conclusions

No clear conclusions can be made from the available data, but early results are promising. Our retrospective study showed that Cyberknife^®^ focal reirradiation is feasible for isolated prostate cancer after radiation therapy fails. It offers excellent results in the field tumor control and low side-effect profile. It is important to note that no severe GU or GI complications were encountered in this cohort despite having had previous high dose of radiation therapy. For highly selected patients, prostate reirradiation using SBRT is a suitable option to treat recurrence after definitive radiation therapy and shows a low toxicity profile. Our data suggest that reirradiation with high dose hypo fractionation may be a rational salvage approach. We also noted an excellent tolerance profile to a patient who received salvage reirradiation despite the high initial dose that patient has received. Prospective study and long term follow up are needed to confirm these findings and identify eligibility criteria for reirradiation therapy.
